# Etiological Subtypes of Transient Ischemic Attack and Ischemic Stroke in Chronic Kidney Disease: Population-Based Study

**DOI:** 10.1161/STROKEAHA.120.030045

**Published:** 2020-08-19

**Authors:** Dearbhla M. Kelly, Linxin Li, Peter M. Rothwell

**Affiliations:** Wolfson Center for Prevention of Stroke and Dementia, Nuffield Department of Clinical Neurosciences, John Radcliffe Hospital, University of Oxford, United Kingdom.

**Keywords:** glomerular filtration rate, hypertension, kidney diseases, stroke, transient ischemic attack

## Abstract

**Background and Purpose:**

Chronic kidney disease (CKD) is strongly associated with stroke risk, but the mechanisms underlying this association are unclear and might be informed by subtype-specific analyses. However, few studies have reported stroke subtypes in CKD according to established classification systems, such as the TOAST (Trial of ORG 10172 in Acute Stroke Treatment) criteria. We, therefore, aimed to determine which transient ischemic attack and ischemic stroke subtypes using the TOAST classification occur most frequently in patients with CKD.

**Methods:**

In a population-based study of all transient ischemic attack and stroke (OXVASC [Oxford Vascular Study]; 2002–2017), all ischemic events were classified by TOAST subtypes (cardioembolism, large artery disease, small vessel disease, undetermined, multiple, other etiology, or incompletely investigated). Logistic regression was used to determine the relationship between CKD (defined as an estimated glomerular filtration rate <60 mL/min per 1.73 m2) and transient ischemic attack/stroke subtypes adjusted for age, sex, and hypertension and then stratified by age and estimated glomerular filtration rate category.

**Results:**

Among 3178 patients with transient ischemic attack (n=1167), ischemic stroke (n=1802), and intracerebral hemorrhage (n=209), 1267 (40%) had CKD. Although there was a greater prevalence of cardioembolic events (31.8% versus 21.2%; *P*<0.001) in patients with CKD, this association was lost after adjustment for age, sex, and hypertension (adjusted odds ratio=1.20 [95% CI, 0.99–1.45]; *P*=0.07). Similarly, although patients with CKD had a lower prevalence of small vessel disease (8.8% versus 13.6%; *P*<0.001), undetermined (26.1% versus 39.4%; *P*<0.001), and other etiology (1.0% versus 3.6%; *P*<0.001) subtypes, these associations were also lost after adjustment (adjusted odds ratio=0.86 [0.65–1.13]; *P*=0.27 and 0.73 [0.36–1.43]; *P*=0.37 for small vessel disease and other defined etiology, respectively) for all but undetermined (adjusted odds ratio=0.81 [0.67–0.98]; *P*=0.03).

**Conclusions:**

There were no independent positive associations between CKD and specific TOAST subtypes, which suggest that renal-specific risk factors are unlikely to play an important role in the etiology of particular subtypes. Future studies of stroke and CKD should report subtype-specific analyses to gain further insights into potential mechanisms.

## Abstract 2

Supplemental Digital Content is available in the text.

As an established cardiovascular risk factor, chronic kidney disease (CKD) is a rising global health burden with prevalence rates of between 11% and 13%.^1,2^ It has been associated with a 43% increased risk of incident stroke,^3^ greater disability and likelihood of institutionalization,^4^ and higher short- and long-term mortality poststroke.^5,6^

Although CKD appears to be a risk factor for stroke analogous to diabetes mellitus,^7^ the mechanisms underpinning this relationship are unclear. Previous meta-analyses have suggested that the relationship between CKD and stroke risk is independent of conventional cardiovascular risk factors frequently comorbid with CKD, such as hypertension, diabetes mellitus, and atrial fibrillation (AF).^3,8^ Proposed causal mechanisms include chronic inflammation, oxidative stress, or thrombogenic factors induced by the uremic milieu that subsequently contribute to vascular injury and endotheliopathy.^9^ In particular, CKD has been associated with increased left atrial thrombogenic milieu in patients with AF,^10^ heavily calcified and unstable carotid plaque morphology in large artery disease,^11^ and uremic disruption of blood-brain barrier integrity^12^ which may have implications for small vessel disease and lacunar stroke risk.^13^

Etiological classification of stroke into different causative subtypes, like the widely used TOAST (Trial of ORG 10172 in Acute Stroke Treatment) system^14^ can provide mechanistic insights as studies have shown that different stroke subtypes may represent different risk factor profiles.^15,16^ The heterogenous pathophysiology of stroke is further highlighted by recent genome-wide association studies that have identified different genetic loci associated with specific stroke subtypes, reflecting differing causal pathways.^17,18^

However, very few studies to date have reported the frequency of the various etiological stroke subtypes that occur in CKD, reporting only the risk of all-type events or ischemic versus hemorrhagic strokes. Those that have subtyped in more detail variably suggest a preponderance of cardioembolic, large vessel, or lacunar events.^19–21^ However, these studies have been small and did not report adjusted risk estimates. CKD is strongly related to age and other risk factors. Our recent meta-analysis has demonstrated that the association between CKD and stroke appears to be highly dependent on the method of adjustment for hypertension, implicating long-term blood pressure burden as the primary confounder of this relationship.^22^

In a large prospective population-based study, we aimed to determine which transient ischemic attack (TIA) and stroke subtypes occur most frequently in patients with CKD, and whether any associations present remained after adjustment for potential confounders.

## Methods

### Data Availability

Requests for access to data should be submitted for consideration to the OXVASC (Oxford Vascular Study) Study Director (peter.rothwell@ndcn.ox.ac.uk).

### Patients

The OXVASC is an ongoing population-based study of all acute vascular events (including TIA, stroke, acute coronary syndromes, and peripheral vascular events) since 2002. The study population comprises all 92 728 individuals, irrespective of age, registered with about 100 general practitioners in 9 general practices in Oxfordshire, United Kingdom. The OXVASC population is 94% White, 3% Asian, 2% Chinese, and 1% Afro-Caribbean.^23^ The methodology of OXVASC was approved by the Oxfordshire Research Ethics Committee. Multiple methods of ascertainment are used to ascertain patients with TIA or stroke, as detailed elsewhere.^24^ Briefly, multiple overlapping methods of hot and cold pursuit are used to achieve near-complete ascertainment of all individuals with TIA or stroke. These include a daily, rapid access TIA clinic to which participating general practitioners and the local emergency department refer all individuals with unhospitalized TIA or stroke; daily searches of ward admissions (medical, cardiology, stroke unit, and neurology), emergency department attendance register and in-hospital bereavement office death records; and monthly searches of death certificates, coroner’s reports (for out-of-hospital deaths), general practitioner and hospital diagnostic/discharge codes, and brain/vascular imaging referrals.

Patients with incident TIA and stroke recruited from April 2002 to March 2017 were included in this analysis. All patients provided written informed consent or assent was obtained from relatives, and they were seen by study physicians as soon as possible after their initial presentation. A detailed clinical history was recorded in all patients using a standardized questionnaire. Neurological impairment, medical history, and risk factors were recorded in all patients. Hypertension was defined on the basis of a historical diagnosis (either patient-reported or general practitioner-coded) or the presence of antihypertensive treatment. Patients routinely had brain imaging, vascular imaging, 12-lead ECG, and standard blood tests. However, during the 15-year study period of OXVASC, different imaging protocols were used in 2 different time periods. From April 1, 2002 to March 31, 2010 (phase 1), computed tomography brain and carotid doppler were the first-line imaging methods, with magnetic resonance imaging or magnetic resonance angiography done in selected patients when clinically indicated. Echocardiography, 24-hour ECG (Holter monitor), and 5-day ambulatory home ECG monitoring (R test) were also done when clinically indicated (eg, potential cryptogenic TIA/stroke; multiterritory infarct; patients at high risk of endocarditis, with known valve problems, or with other cardiological complaints). From April 1, 2010 to March 31, 2017 (phase 2), brain magnetic resonance imaging and magnetic resonance angiography of extracranial and intracranial vessels became the first-line imaging methods, and all clinic patients had R tests and transthoracic echocardiography.

Although new definitions for stroke and TIA have been suggested recently,^25,26^ to enable comparison with previous studies, the classic definitions of TIA and stroke are used throughout.^26^ A stroke is defined as rapidly developing clinical symptoms and signs of focal, and at time global (applied to patients in deep coma and to those with subarachnoid hemorrhage), loss of brain function, with symptoms lasting >24 hours or leading to death, with no apparent cause other than that of vascular origin.^27^ A TIA is an acute loss of focal brain or monocular function with symptoms lasting <24 hours and which is thought to be caused by inadequate cerebral or ocular blood supply as a result of arterial thrombosis, low flow, or embolism associated with arterial, cardiac, or hematological disease.^23^ All cases were reviewed by a senior neurologist (Dr Rothwell) daily, and imaging was reviewed by the study neuroradiologist. All patients were followed up by a research nurse or physician at 1, 3, 6, 12, 24, 60, and 120 months after the index event.

CKD was defined as estimated glomerular filtration rate (eGFR) <60 mL/min per 1.73 m^2^ for 3 or more months as per 2012 Kidney Disease: Improving Global Outcomes guidelines.^28^ eGFR was estimated using the CKD-Epidemiology Collaboration Equation. eGFR was then categorized into 5 groups based on modified CKD classification by the National Kidney Foundation-Kidney Disease Outcomes Quality Initiative: eGFR≥90 (reference), 60 to 89, 30 to 59, 15 to 30, and <15 mL/min per 1.73 m^2^. For the purpose of statistical analysis, the latter 2 groups were combined as the individual numbers within each group were small.

All clinical history and investigation results were reviewed in detail by a study physician using a standardized form (Materials in the Data Supplement) as soon as completion of all investigations, and cases were then reviewed with a senior neurologist (Dr Rothwell) and TIA/ischemic stroke cause was classified (blind to CKD status) according to the modified TOAST criteria into 7 subtypes: cardioembolic stroke, large artery disease, small vessel disease, undetermined cause, unknown etiology, multiple causes, or other defined cause.^14^ We have previously described the specific criteria used to diagnose each TOAST subtype in detail.^29^ The patients were classified as undetermined stroke only if the diagnostic work-up included at least brain imaging, ECG, and carotid imaging, and no clear cause was found. Patients with more incomplete investigations were classified as unknown stroke while stroke of multiple causes was classified separately. Although TOAST criteria have not been specifically validated for TIA events, its practical application in a TIA cohort has been previously demonstrated with comparable results to other classification systems.^30^

### Statistical Analysis

Descriptive statistics were used to summarize the baseline characteristics of the cohort stratified by CKD status. Continuous data were given as mean (SD) or median (interquartile range) as appropriate, categorical data were given as n (%). Mann-Whitney *U* and χ^2^ tests were used to test the significance of differences between 2 groups for continuous and categorical variables, respectively. The relative frequency of TOAST subtypes was reported according to CKD status. Associations between CKD and TOAST subtypes were determined by binary logistic regression whereby specific subtypes were treated as dichotomous dependent variables and compared with all other subtypes, adjusted for age, sex, and hypertension, and stratified by age (<65 versus ≥65 years) and eGFR category. Statistical heterogeneity in CKD prevalence among TOAST subtypes was investigated using metaregression to determine if age was a significant predictor of between-subtype variance. Logistic regression analysis was also used to determine the association between ICH and CKD using ischemic stroke as the reference category. Events of unknown etiology (ie, incompletely investigated) were excluded from all regression analyses to avoid the risk of reverse causation (ie, under-investigation of elderly, frail patients with CKD due to contraindications or frailty). Results were considered significant at *P*<0.05. All statistical analyses were performed using SPSS version 25.0.

## Results

A total of 3178 consecutive eligible patients with TIA (n=1167), ischemic stroke (n=1802), and ICH (n=209) were recruited from 2002 to 2017. Table 1 shows the baseline characteristics at the time of the event for all TIA/ischemic stroke patients and according to CKD status. The median age was 75.8 (66.0–83.7) years, 49.1% (n=1458) were men, and hypertension was the most prevalent risk factor being found in 58.5% (n=1738). The median eGFR was 65.9 mL/min per 1.73 m^2^, and 1197 patients had CKD (40.3% of the study population). A total of 422 patients (14.2%) had an eGFR≥90, 1346 (45.3%) had an eGFR 60 to 89, 1064 (35.8%) had an eGFR 30 to 59, 133 (4.5%) had an eGFR<30 mL/min per 1.73 m^2^. Only 11 patients (0.3%) were dialysis dependent. Notably, compared with those with normal renal function, the CKD group was older and had a significantly higher burden of vascular risk factors and comorbidities, including hypertension, diabetes mellitus, ischemic heart disease, peripheral arterial disease, congestive cardiac failure, and AF (all *P*<0.05).

**Table 1.**
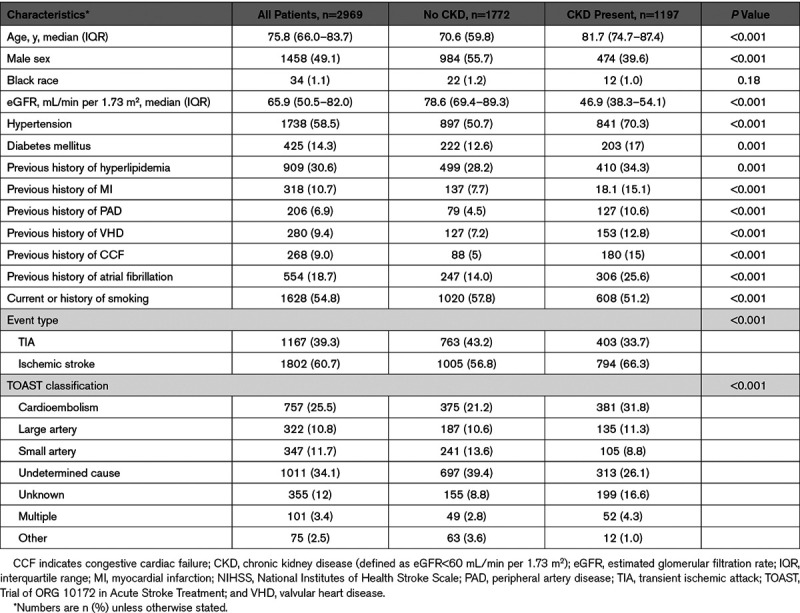
Baseline Characteristics of All Patients With TIA and Ischemic Stroke, and Stratified According to the Presence of CKD

**Figure 1.**
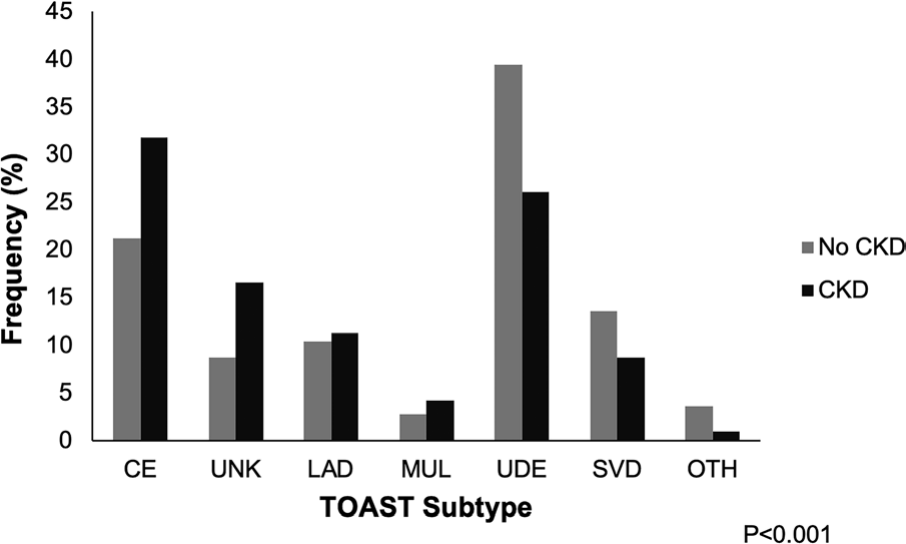
**Relative frequencies of TOAST (Trial of ORG 10172 in Acute Stroke Treatment) transient ischemic attack/stroke subtypes according to chronic kidney disease (CKD) status.** CE indicates cardioembolism; LAD, large artery disease; MUL, multiple causes; OTH, other causes; SVD, small vessel disease; UDE, undetermined; and UNK, unknown.

Table 1 and Figure 1 demonstrate the relative frequency of TIA/ischemic stroke TOAST subtypes occurring with the entire population and according to the presence or absence of CKD. There was a greater prevalence of cardioembolic subtype (31.8% versus 21.2%; *P*<0.001), unknown (16.6% versus 8.8%; *P*<0.001), and multiple cause (4.3% versus 2.8%; *P*=0.03) events in patients with CKD while small vessel disease (8.8% versus 13.6%; *P*<0.001), undetermined (26.1% versus 39.4%; *P*<0.001), and other etiologies (1.0% versus 3.6%; *P*<0.001) were more common in patients without CKD. There was no significant difference in large artery disease subtype prevalence between groups (11.3% versus 10.6%; *P*=0.59).

**Figure 2.**
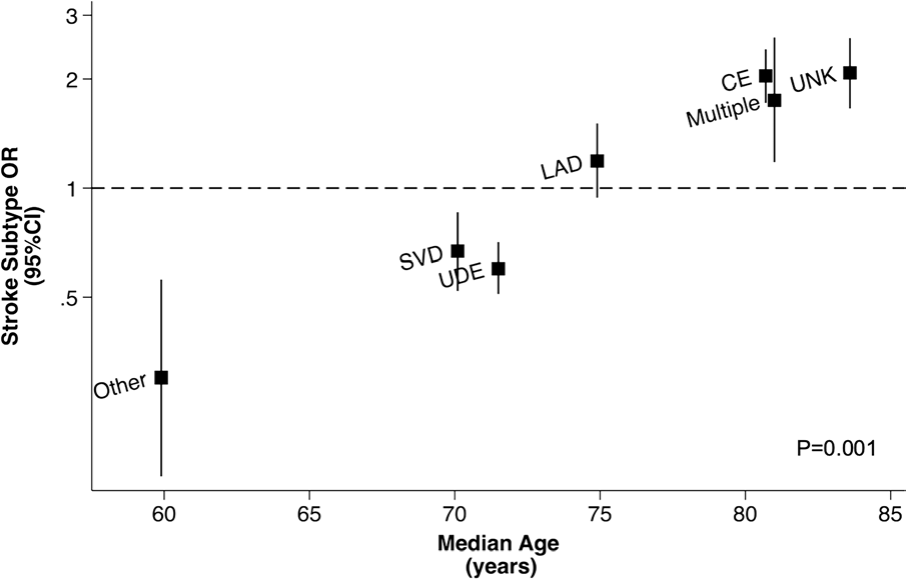
**The odds ratio (OR) of specific TOAST (Trial of ORG 10172 in Acute Stroke Treatment) subtypes in chronic kidney disease (CKD) vs the median age within individual subtypes.** CE indicates cardioembolism; LAD, large artery disease; SVD, small vessel disease; UDE, undetermined; and UNK, unknown.

**Figure 3.**
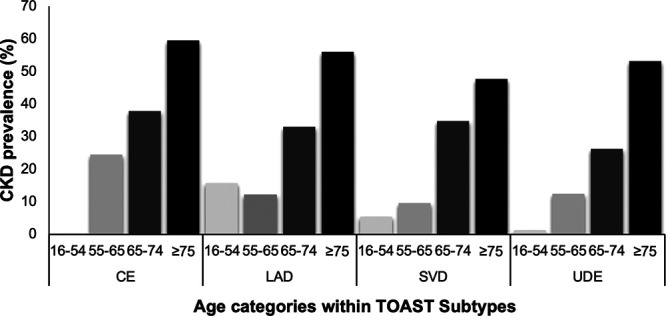
**Chronic kidney disease (CKD) prevalence within TOAST (Trial of ORG 10172 in Acute Stroke Treatment) subtypes according to age category.** CE indicates cardioembolism; LAD, large artery disease; SVD, small vessel disease; and UDE, undetermined.

Although there appeared to be major differences in CKD prevalence between TIA/stroke subtypes (*P*<0.001; Figure 1), CKD prevalence was also strongly associated with age. When the median age of each TIA/stroke subtype was plotted against the odds ratio (OR) of individual subtypes, there was a linear association suggesting potential confounding by age (*P* value for heterogeneity=0.001; Figure 2). Consistent with this hypothesis, the prevalence of CKD showed a similar pattern of variation according to age within individual TIA/stroke subtypes (Figure 3).

The association between CKD and TOAST subtypes was further examined using univariate and multivariate regression analysis (Table 2). In unadjusted analysis, CKD appeared to be associated with a significantly increased risk of cardioembolic TIA/stroke (crude OR=2.04 [95% CI, 1.72–2.42]; *P*<0.001) and events of multiple etiologies (crude OR=1.75 [1.18–2.61]; *P*=0.006). However, these risk associations attenuated and became nonsignificant after adjustment for age, sex, and hypertension (adjusted OR=1.20 [0.99–1.45]; *P*=0.07 for cardioembolic events and adjusted OR=1.13 [0.73–1.74]; *P*=0.59 for events of multiple causes). CKD initially appeared to be associated with lower risk of small vessel disease (crude OR=0.67 [0.52–0.86]; *P*=0.001), events of undetermined cause (crude OR=0.60 [0.51–0.71]; *P*<0.001), and events of other defined etiology (crude OR=0.30 [0.16–0.56]; *P*<0.001), but again these associations were lost after adjustment for age, sex, and hypertension (adjusted OR=0.86 [0.65–1.13]; *P*=0.27 for small vessel disease events and adjusted OR=0.73 [0.37–1.45]; *P*=0.36 for events of other defined cause), apart from events of undetermined etiology which remained significantly negatively associated with CKD (adjusted OR=0.81 [0.67–0.98]; *P*=0.03). There was no overall association between CKD and large artery disease in either unadjusted or adjusted analysis. Additional adjustment for any temporal trend in CKD prevalence over time did not alter the findings.

**Table 2.**
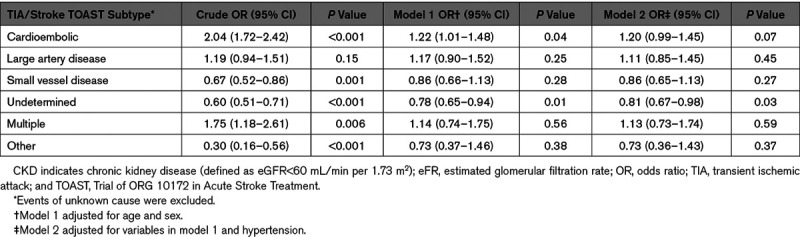
Associations of CKD and TOAST Subtypes, Adjusted for Age, Sex, and Hypertension

Table I in the Data Supplement shows the crude and adjusted ORs of TOAST subtypes according to eGFR categories. After adjustment for age, sex, and hypertension, there were no independent associations between any of the eGFR categories and specific TOAST subtypes.

The age-specific associations of CKD and TOAST subtypes are shown in Table 3. CKD was associated with a significantly increased risk of cardioembolic events in patients <65 years even after adjustment for age, sex, and hypertension (adjusted OR=1.99 [1.02–3.82]; *P*=0.04) but not at older ages (adjusted OR=1.10 [0.90–1.35]; *P*=0.35). There was a nonsignificant association between CKD and large artery disease events in those aged <65 years (adjusted OR=1.32 [0.62–2.82]; *P*=0.47). With further analysis though, there was an independent association between CKD and large artery disease in those aged <55 years (adjusted OR=6.20 [1.18–32.51]; *P*=0.03; Figure 3). There was also a significant inverse association between CKD and other causes in older patients (adjusted OR=0.40 [0.17–0.96]; *P*=0.04).

**Table 3.**
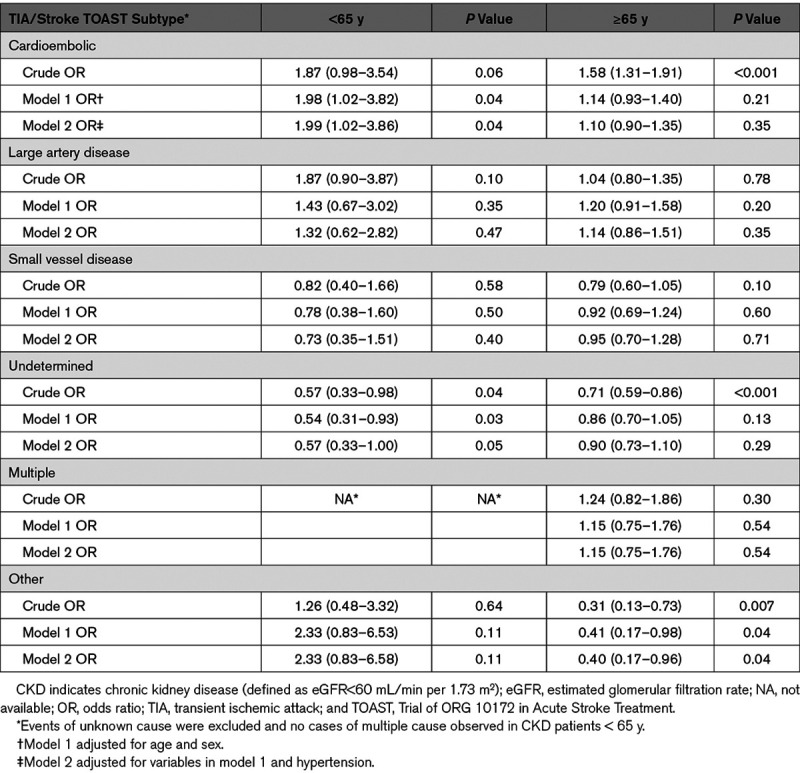
The Age-Specific Associations of CKD and TOAST Subtypes, Adjusted for Age, Sex, and Hypertension

The baseline characteristics of all patients with ICH are summarized in Table II in the Data Supplement. Of the 208 patients, 70 (33.7%) had CKD. Patients with CKD were older (median age 81 versus 71 years; *P*<0.001), more hypertensive (71.4% versus 43.5%; *P*<0.001), and more likely to have AF (21.4% versus 9.4%; *P*=0.03). Compared with ischemic stroke, there initially appeared to be a lower prevalence of ICH in CKD (unadjusted OR=0.64 [0.48–0.87]; *P*=0.004) but after adjustment for age, sex, and hypertension, there was no association between CKD and ICH (adjusted OR=0.75 [0.54–1.05]; *P*=0.09).

## Discussion

Using a population-based cohort study, we report for the first time the relative frequency of TIA and stroke subtypes in CKD using the TOAST classification.^14^ Although there was a greater prevalence of cardioembolic, large artery disease, and multiple etiology subtypes in the CKD population, attenuation of any associations present with adjustment for mainly age but also hypertension is consistent with an earlier stroke risk meta-analysis,^22^ suggesting that there are no important renal-specific vascular risk factors beyond these covariates.

Similar to previous studies,^19,21^ cardioembolic events were the most frequent subtype within the CKD group, accounting for 32% of TIA/ischemic stroke events. Patients with CKD are known to be at high risk of AF with prevalence rates of 12% reported and increasing incidence rates with advancing kidney disease stages.^31^ However, as the association between CKD and cardioembolic events was greatly diminished with adjustment for age, sex, and hypertension, this would suggest that despite being linked to a greater left atrial thrombogenic milieu,^10^ CKD itself is not an overall independent risk factor for these events. The notable exception appears to be for younger patients whom the association remained significant even with adjustment, suggesting possible synergy with other underlying risk factors such as female sex and hypertension in this subgroup. However, the addition of renal function to existing stroke risk prediction tools in AF (eg, CHADS_2_ or CHA_2_DS_2_-VASc) has not been shown to independently add to the predictive value of these scores,^32^ although this may also be a reflection of the accuracy of such scores in a group where their use has not been validated.

Although low eGFR has been consistently associated with subclinical small vessel disease, particularly silent cerebral infarction,^33,34^ we report a lower frequency of symptomatic lacunar TIA/stroke events in the CKD population in this study. There was, in fact, no association between CKD and small vessel disease events after adjustment for age and sex. However, our findings are in keeping with an earlier systematic review and meta-analysis that also did not find any specific association between CKD and symptomatic lacunar stroke, but that in those without stroke, greater small vessel disease burden on imaging was associated with worse renal function.^35^ The authors postulated that small vessel disease events may be underestimated in this group due to imprecise subtyping with an overreliance on clinical and computed tomography diagnosis. Similarly, in our study, not all patients would have had diffusion-weighted imaging-magnetic resonance imaging in the acute phase, particularly those with more advanced CKD, which may limit the sensitivity of our lacunar stroke subtyping. Previous studies have also suggested that there may be age-specific associations between small vessel disease (either markers or strokes) and CKD,^35,36^ however, we did not any differential risk association between those aged >65 or <65 years old.

Although there was no overall association between CKD and large artery disease events, there did appear to be an age-specific association present for those aged <55 years. There are a number of potential mechanisms that may underlie this relationship. Renal function has been shown to be a strong predictor of greater carotid intima-media thickness and progression of subclinical atherosclerosis independent of traditional and nontraditional cardiovascular risk factors.^37^ Dialysis appears to accelerate medial vascular calcification in children and young people,^38^ and blood vessels from children with CKD show features of premature aging, including oxidative DNA damage and elevated senescence markers.^39^ There is also evidence of polygenic correlation between CKD and large artery disease stroke.^40^ However, the total number of stroke events among younger people in this category was quite low so further research in this area is required.

CKD was associated with a significantly lower risk of TIA/stroke events of undetermined etiology. Since cryptogenic TIA/strokes have been associated with few traditional atherosclerotic or cardioembolic markers,^29^ this finding is in keeping with our hypothesis that most of the stroke risk in kidney disease may be accounted for by age and hypertension.^22^ If nontraditional risk factors such as inflammation, oxidative stress, or coagulopathy related to the uremic milieu were etiologically important, one might have expected a higher burden of events of undetermined cause in patients with CKD, as these factors are not measured in conventional TIA/stroke work-up and may be better represented by this subtype.

Similar to previous studies,^41^ CKD was present in one-third of patients with ICH. However, there was no specific association between ICH and CKD as compared to ischemic stroke risk. This is consistent with earlier work that suggests that proteinuria associates more strongly with hemorrhagic stroke risk than low eGFR.^42,43^ It may also be a reflection of the underlying predominantly White population as the relationship between CKD and ICH appears to be stronger in Asian^44^ and Black populations, possibly attributable to a higher presence and number of cerebral microbleeds in the latter.^45^

Our study had a number of limitations. First, there are shortcomings with all etiological classifications of TIA/ischemic stroke. Although the TOAST classification is the most widely used system, there may be multiple different pathologies within each of the TOAST subcategories, particularly strokes of undetermined etiology. Second, cryptogenic events (undetermined and unknown subtypes) accounted for a large proportion of cases, limiting mechanistic insights. However, this is consistent with other large epidemiological studies where cryptogenic strokes accounted for 26% to 40% of cases.^46,47^ Third, since a minority of patients did not have intracranial vessel imaging or brain magnetic resonance imaging, it is possible that there was misclassification bias whereby large artery disease and small vessel disease events were misclassified as undetermined. The changes to diagnostic evaluation over time could have resulted in differences in subtype designation through the study period. Fourth, lack of association between CKD and specific TOAST subtypes after adjustment for age and hypertension does not necessarily indicate that renal-specific risk factors do not play a role in the cause of all stroke. We can only conclude that any such role does not differ in importance between the different etiological subtypes. Fifth, direct comparison of individual subtype-CKD associations in a case only setting can be challenging given that each subtype group is then included in the referent group of the other. Finally, as previously stated, the OXVASC population study is 94% White, which may limit the generalizability of our results to other settings.

However, this study has broader implications for stroke research. It highlights how any study of risk association and stroke should be carefully stratified by and adjusted for age. Previous studies that reported CKD prevalence according to stroke subtype did not adjust for age,^19–21^ and as evidenced by this article, subtype-specific associations are particularly prone to confounding by age.

In conclusion, to the best of our knowledge, this was the first study to subtype in detail TIA/stroke events in patients with CKD according to the TOAST classification. We found few associations between CKD and specific event subtypes after adjustment for age, sex, and hypertension suggesting that addressing traditional risk factors may be most important in terms of prevention and treatment.

## Acknowledgments

We are grateful to all the staff in the general practices that collaborated in the OXVASC (Oxford Vascular Study): Abingdon Surgery, Stert St, Abingdon; Malthouse Surgery, Abingdon; Marcham Road Family Health Centre, Abingdon; The Health Centre, Berinsfield; Key Medical Practice; Kidlington; 19 Beaumont St, Oxford; East Oxford Health Centre, Oxford; Church Street Practice, Wantage. We also acknowledge the use of the facilities of the Acute Vascular Imaging Centre, Oxford.

## Sources of Funding

Dr Rothwell has received funding from Wellcome Trust, Wolfson Foundation, British Heart Foundation, National Institute for Health Research, and the National Institute for Health Research Oxford Biomedical Research Centre and has received payment for membership of a randomized trial Executive Committee (Bayer). D.M. Kelly has received a scholarship from the Irish Nephrology Society.

## Disclosures

None.

## Supplementary Material


