# Supplementary material for: Etiological Subtypes of Transient Ischemic Attack and Ischemic Stroke in Chronic Kidney Disease: Population-Based Study
Source: Stroke. 2020 Aug 19;51(9):2786–94. doi: 10.1161/STROKEAHA.120.030045 (PMC7447187; doi:10.1161/STROKEAHA.120.030045)
Supplement: Supplementary file 1 [file str-51-2786-s001.pdf]

ONLINE SUPPLEMENTAL MATERIALS

Aetiological subtypes of TIA and ischaemic stroke in chronic kidney disease: population-based Study

Dearbhla M. Kelly, MBBChBAO MSc MRCPI,[1] Linxin Li, DPhil,[1] Peter M. Rothwell MD PhD FRCP FMedSci[1] on behalf of the Oxford Vascular Study.

[1]Wolfson Center for Prevention of Stroke and Dementia, Nuffield Department of Clinical Neurosciences, John Radcliffe Hospital, University of Oxford, United Kingdom.

Corresponding author:

Professor Peter Rothwell,

Wolfson Center for Prevention of Stroke and Dementia, Nuffield Department of Clinical Neurosciences, John Radcliffe Hospital, University of Oxford, United Kingdom.

Tel no: +441865231601

Email: peter.rothwell@ndcn.ox.ac.uk

TABLE OF CONTENTS

Page Number

TOAST Classification for OXVASC……………………………………………………..3-4

Supplementary Tables

Table I: Associations of GFR categories and TOAST subtypes, adjusted for age and hypertension…………………………………………………………………………..5

Table II: Baseline characteristics of all patients with ICH, and stratified according to the presence of CKD…………………………………………………………..6

TOAST for OXVASC

Patient Name: **Event Date :**

OXVASC ID: **Event Type :**

Age at event:

Risk factor profile (for **all patients**; please circle)

Cardioembolic-related:

Diagnosed AF (including atrial flutter) prior to the index event: Yes / No
Mechanical prosthetic /Bioprosthetic cardiac valve prior to the index event: Yes / No

If yes to either→Treatment: anticoagulation / antiplatelet / none INR (if on warfarin) ______

Recent MI (<4 weeks): Yes / No

Atherosclerotic risk factors:

Hypertension / DM / hyperlipidaemia / myocardial infarction / PVD / stroke / TIA / smoking (ex/current).

Other risk factors:

None / Migraine with aura / Migraine without aura / Autoimmune disease/ Active cancer

Clinical syndrome (for **all patients;** please circle)

NINDS criteria (PMR): Positive / Negative

Clinical syndrome according to the OXVASC classification (PMR):

OCSP: PACI / LACI / TACI / POCI

Vascular territory: LACA / LMCA / LPCA / RACA / RMCA / RPCA / VB/uncertain

Investigations (**ONLY** for **prob/def patients**; please circle)

Brain imaging **CT / MRI / Both**

Acute lesion Yes / No If Yes, please circle the appropriate description below:

Single: L / R, Size _____ mm, Carotid / VB, Cortical / Subcortical

lacunar infarct Yes / No

Multiple: L / R / Both; Anterior / Posterior / Both

Old lesion Yes / No If Yes, please describe ______________________________________

Vascular imaging **Carotid Doppler only**

Normal Yes / No

Dissection Yes / No, if Yes, please describe ____

Stenosis Yes / No If Yes, please circle the appropriate description below:

Symptomatic / asymptomatic / Both

If symptomatic, please choose the location below:

Extracranial / Intracranial l / Both, Carotid / VB / Both

Severity ($\geq$ 50%) Yes / No

Known stenosis from previous scans (Doppler/CTA/MRA/DSA)? No / Yes, please specify_________

Bubble TCD R-L shunt Yes / No

Cardiac investigation

ECG: SR / AF (including atrial flutter) / sick-sinus syndrome / other: _____________

Echo: EF _____ %

Left ventricular or Left atrial / atrial appendage thrombus	Yes / No
Akinetic / hypokinetic left ventricular segment	Yes / No
Mitral stenosis	Yes / No
Mitral valve prolapse	Yes / No
Infective / non-bacterial thrombotic endocarditis	Yes / No
PFO / Atrial septum aneurysm	Yes / No
Myxoma	Yes / No

R-test/HOLTER: AF>30s Yes / No

Other investigation results ____

(*e.g.* thrombophilia screen, CADASIL genetics)

TOAST (**ONLY** for **prob/def patients;** please circle)

LAD / CE / SVD / UDE / UNK / MULT_______________/ Other___________________

OXCODE (for **all patients**)

Supplementary Table I: Associations of eGFR categories and TOAST subtypes, adjusted for age, sex, and hypertension.

	GFR Categories (ml/min/1.73m^2)						
**TIA/Stroke TOAST Subtype†**	**≥90**	**60-89**		**30-59**		**<30**	
**Cardioembolic**			**P value**		**P value**		**P value**
Crude OR	1.00 (ref)	1.82 (1.35-2.45)	<0.001	3.08 (2.28-4.15)	<0.001	5.50 (3.38-8.96)	<0.001
Model 1 OR*	1.00 (ref)	0.91 (0.65-1.28)	0.58	0.93 (0.62-1.41)	0.74	2.61 (1.33-5.10)	0.01
Model 2 OR**	1.00 (ref)	0.91 (0.65-1.29)	0.60	0.91 (0.60-1.38)	0.67	2.54 (1.30-4.97)	0.01
**Large artery disease**							
Crude OR	1.00 (ref)	1.20 (0.83-1.74)	0.32	1.39 (0.96-2.03)	0.08	1.19 (0.59-2.42)	0.63
Model 1 OR	1.00 (ref)	0.91 (0.60-1.39)	0.67	1.76 (1.03-3.01)	0.04	0.85 (0.33-2.15)	0.73
Model 2 OR	1.00 (ref)	0.92 (0.60-1.40)	0.70	1.62 (0.95-2.77)	0.08	0.83 (0.33-2.11)	0.70
**Small vessel disease**							
Crude OR	1.00 (ref)	0.76 (0.56-1.03)	0.07	0.57 (0.41-0.79)	0.001	0.32 (0.14-0.77)	0.01
Model 1 OR	1.00 (ref)	0.94 (0.66-1.33)	0.73	1.15 (0.70-1.88)	0.59	0.47 (0.17-1.29)	0.15
Model 2 OR	1.00 (ref)	0.94 (0.67-1.33)	0.73	1.18 (0.72-1.93)	0.53	0.46 (0.17-1.26)	0.13
**Undetermined**							
Crude OR	1.00 (ref)	0.77 (0.61-0.96)	0.02	0.51 (0.40-0.65)	<0.001	0.32 (0.19-0.54)	<0.001
Model 1 OR	1.00 (ref)	1.10 (0.85-1.43)	0.46	0.81 (0.57-1.15)	0.23	0.38 (0.20-0.72)	0.003
Model 2 OR	1.00 (ref)	1.10 (0.85-1.43)	0.47	0.84 (0.59-1.20)	0.33	0.39 (0.21-0.76)	0.01
**Multiple**							
Crude OR	1.00 (ref)	1.49 (0.72-3.09)	0.29	2.33 (1.13-4.81)	0.02	3.04 (1.05-8.76)	0.04
Model 1 OR	1.00 (ref)	0.64 (0.27-1.47)	0.29	0.59 (0.23-1.55)	0.26	0.90 (0.19-4.18)	0.89
Model 2 OR	1.00 (ref)	0.63 (0.27-1.47)	0.29	0.58 (0.22-1.50)	0.26	0.87 (0.19-3.99)	0.86
**Other**							
Crude OR	1.00 (ref)	0.60 (0.36-1.03)	0.06	0.21 (0.10-0.44)	<0.001	0.19 (0.03-1.42)	0.11
Model 1 OR	1.00 (ref)	1.57 (0.85-2.88)	0.15	0.97 (0.34-2.77)	0.95	0.89 (0.10-8.11)	0.91
Model 2 OR	1.00 (ref)	1.56 (0.85-2.88)	0.15	0.95 (0.33-2.74)	0.93	0.92 (0.10-8.47)	0.94

eGFR indicates estimated glomerular filtration rate; OR, odds ratio.
†Events of unknown aetiology were excluded.
*Model 1 adjusted for age and sex.
**Model 2 adjusted for variables in model 1 and hypertension.

Supplementary Table II: Baseline characteristics of all patients with ICH, and stratified according to the presence of CKD

Characteristics*	All patients n= 209	No CKD n= 138	CKD present n= 70	P value
Age years, median (IQR)	76 (61.5-83.0)	71 (56.8-80.3)	81 (75.0-88.0)	<0.001
Male sex	103 (49.3)	71 (51.4)	32 (45.7)	0.53
Black race	3 (1.4)	3 (2.2)	0 (0)	0.53
eGFR (ml/min/1.73m^2), median (IQR)	72.3 (53.1-87.4)	83.4 (72.2-93.1)	47.5 (39.7-53.3)	<0.001
Hypertension	110 (52.6)	60 (43.5)	50 (71.4)	<0.001
Diabetes mellitus	24 (11.5)	12 (8.7)	12 (17.1)	0.12
Previous history of hyperlipidaemia	43 (20.6)	26 (18.8)	17 (24.3)	0.46
Previous history of MI	5 (2.4)	4 (2.9)	1 (1.4)	0.86
Previous history of PAD	9 (4.3)	5 (3.6)	4 (5.7)	0.73
Previous history of atrial fibrillation	28 (13.4)	13 (9.4)	15 (21.4)	0.03
Current or history of smoking	99 (49)	70 (52.2)	29 (43.3)	0.30

*Numbers are n (%) unless otherwise stated.

CCF indicates congestive cardiac failure; CKD, chronic kidney disease (defined as eGFR<60 mL/min per 1.73 m^2); eGFR, estimated glomerular filtration rate ICH indicates intracerebral haemorrhage, IQR indicates interquartile range; MI, myocardial infarction; NIHSS, National Institutes of Health Stroke Scale; PAD, peripheral artery disease; TIA, transient ischaemic attack; VHD, valvular heart disease;
